# Time Course of Motor Sleep Inertia Dissipation According to Age

**DOI:** 10.3390/brainsci12040424

**Published:** 2022-03-23

**Authors:** Lorenzo Tonetti, Marco Fabbri, Sara Giovagnoli, Monica Martoni, Miranda Occhionero, Vincenzo Natale

**Affiliations:** 1 Department of Psychology “Renzo Canestrari”, University of Bologna, 40127 Bologna, Italy; sara.giovagnoli@unibo.it (S.G.); miranda.occhionero@unibo.it (M.O.); vincenzo.natale@unibo.it (V.N.); 2 Department of Psychology, University of Campania Luigi Vanvitelli, 81100 Caserta, Italy; marco.fabbri@unicampania.it; 3 Department of Experimental, Diagnostic and Specialty Medicine, University of Bologna, 40138 Bologna, Italy; monica.martoni@unibo.it

**Keywords:** sleep inertia, motor activity, actigraphy, sleep, age

## Abstract

Sleep inertia (SI) refers to a complex psychophysiological phenomenon observed after morning awakening that can be described as the gradual recovery of waking-like status after a night of sleep. The time course of SI dissipation in an everyday life condition is little studied. The present study aims to investigate the SI dissipation in motor activity, as a function of age, upon spontaneous morning awakening after a usual night-time sleep. To this end, we performed a retrospective study in a naturalistic setting in a wide life span sample: 382 healthy participants (219 females) from middle childhood (9 years old) to late adulthood (70 years old). Participants were required to wear the actigraph on the non-dominant wrist for at least seven consecutive nights. Results show that SI of motor activity is dissipated in 70 min. Mean motor activity in such a time window was significantly modulated by age: lower age corresponded to higher motor activity.

## 1. Introduction

Sleep inertia (SI) [1] refers to a complex psychophysiological phenomenon, observed after the last morning awakening, that can be described as the gradual recovery of waking-like status, being defined in the last review on this event as a “transitional state between sleep and wake, marked by impaired performance, reduced vigilance, and a desire to return to sleep” [2], (p. 76). This transition is not based on an “all-or-nothing” process but acts according to slow and progressive mechanisms [2]. Several factors can affect SI, such as the total sleep time or sleep deprivation [3] as well as the sleep stage of awakening [4]. A recent study [5] carried out in a laboratory setting under a polysomnographic control, has investigated the dissipation of SI in different memory domains (i.e., semantic, episodic, and procedural) upon the morning awakening from different sleep stages: REM or NREM-stage 2. While no differences were observed according to the stage of awakening as well as in the episodic or procedural memory systems, SI significantly affected the performance speed but not the accuracy in the semantic memory task. More in detail, a dissipation of SI was observed within 20 min from the NREM-stage 2 awakening, while within 30 min from the REM awakening. Regardless of the SI duration and time course, an even more recent study [6] aimed to detect different subjective SI profiles in the general Chinese population using the latent profile analysis. Authors disclosed four different SI profiles, i.e., low, mild, moderate, and severe, differing, for example, in age and perceived sleep quality. The SI phenomenon also varies according to the method of measurement used: behavioral (cognitive task) [7] or physiological [8]. For this reason, the duration and time course of SI are two of its most controversial aspects [9]. According to a careful analysis of the literature, Åkerstedt and Folkard [10] concluded that complete SI dissipation in alertness takes on average 3 h and twenty-one minutes. Although the biological substrate of SI should still be fully understood, the potential role played by adenosine has been highlighted [11]. In line with this hypothesis, it has been shown that caffeine, an antagonist of adenosine receptors, may alleviate the impairments due to SI [12,13]. Furthermore, it has been proposed a potential association of thermoregulation [14] and hypothalamic-pituitary-adrenal axis [15] with SI, since the time course of extremity cooling and cortisol awakening response resembles the decline of subjective sleepiness after the awakening.

Because SI has been usually tested in particular sleep conditions, such as sleep deprivation or sleep fragmentation, paradoxically, little research has examined SI in a normal sleep-wake cycle condition (i.e., in everyday life). Considering the above-mentioned issues, the main purpose of this research is to ecologically evaluate the SI phenomenon, monitoring motor activity by actigraphy. Being a small device that records motor activity, the actigraph is usually used to assess sleep at home (naturalistic setting) for multiple nights when no sleep stage identification is required [16]. Considering that the actigraph actually measures motor activity, we propose that this measure can be a very useful behavioral index also for evaluating the phenomenon of SI. Indeed, it has been shown that ending sleep in the morning involves a reorganization of brain networks after awakening, including motor areas [17,18]. In particular, the default-mode network (DMN) showed a prompt recovery (within 6 min) after morning awakening, while the full reorganization of the connectivity of the Sensorimotor Network (SMN) was recovered within 30 min [19].

For these reasons, we chose to investigate SI in a naturalistic setting using motor activity, recorded by actigraph, as the dependent variable. The aims of the research are to evaluate: (1) how long it would take to dissipate motor SI after the final awakening in the morning; (2) whether and how age could modulate motor SI dissipation. Since this is the first research to investigate motor SI, we were unable to predict the expected results related to the first goal of the study. Based on knowledge of the cortisol awakening response (CAR), i.e., the rapid rise in cortisol levels observed immediately following awakening, which peaks approximately between 30 and 45 min after awakening [20], and the full reorganization of the brain networks [17], we would expect motor SI to last at least 30 min. Regarding the second aim, we expected that age could modulate the phenomenon of motor SI. In particular, we hypothesized that the SI phenomenon would be more evident, i.e., a greater amplitude, in young people because morning cortisol secretion linearly increases with age in children and the adolescent population [21] while the cortisol concentration shifts in the late evening and early night in older people [22].

Due to the complexity of the sleep phenomenon, the transition between sleep and wake is complex too. Such transition, namely the SI, can be conceived as a multifactorial phenomenon in which different levels of analysis (e.g., hormonal and cognitive) are not always incorporable within a single reference model and cannot always be simultaneously examined. The potential advantage of looking at the SI from a motor point of view, through actigraphy, is the ecological validity of this measure. Other than having an ecological picture of SI, such an approach would also allow for the examination of more awakenings for each participant, reducing the potential effect of intra-individual differences.

## 2. Materials and Methods

### 2.1. Participants

In this research, the participants came from previous studies [23,24,25,26,27,28]. The complete sample consisted of 382 healthy participants (219 females and 163 males) whose ages range from nine to seventy years. The participants were part of a large sample with regular sleep habits, no drug or medication use that could affect sleep and/or cognition. More details on the sample of participants can be found in a previous article [29].

### 2.2. Materials and Procedure

The sleep–wake cycle was monitored using the Actiwatch AW64 actigraph (Cambridge Neurotechnology Ltd., Fenstanton, UK). The participants wore the actigraph on their non-dominant wrist for a mean period of 7.40 ± 2.40 consecutive days for a total of 2826 nights. They were also instructed to push the actigraph event-marker button to signal when they went to bed and woke up in the morning.

The hardware consists of a piezoelectric accelerometer with a sensitivity of ≥0.05 g. The sampling frequency was set to 32 Hz, and filters to 3–11 Hz. The actigraphs were initialized by Actiwatch Activity and Sleep Analysis 5 software (version 5.32, Cambridge Neurotechnology Ltd., Fenstanton, UK) to collect data in 1-min epochs, the standard sampling rate to study sleep [30].

Version 5.32 of the Actiwatch Activity and Sleep Analysis software (Cambridge Neurotechnology Ltd., Fenstanton, UK) was also used to extract the raw motor activity counts, minute-by-minute, considering the hours close to wake-up time (i.e., two hours before and three hours after wake-up time [10]). To avoid the possible confounding weekend effect, for each participant, the mean of the work/school days was computed, allowing us to describe the raw mean motor activity pattern of sleep-wake transition.

### 2.3. Statistical Analyses

Regarding the motor activity pattern of sleep-wake transition, the Functional Linear Modeling (FLM) [31], a statistical framework specifically developed for the analysis of actigraphic data, was used in order to detect if and when the pattern statistically differed according to a continuous variable, i.e., age, over the time interval included between two hours before and three hours after the get-up time.

The raw motor activity pattern of sleep-wake transition was substituted with a function by applying the Fourier expansion model and was then examined through a non-parametric permutation F-test in order to establish a significant relationship between motor activity and age.

## 3. Results

Results of the effect of age on sleep quality, quantity, and timing in this sample have already been published [29], and overall they agree with literature evidence [32].

**Figure 1:**
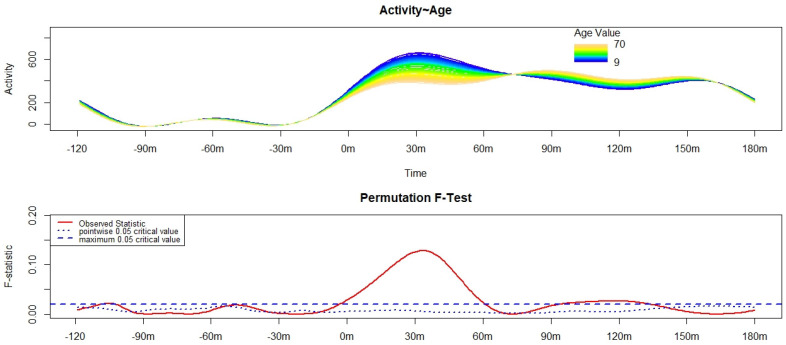
Results of the Functional Linear Modeling applied to the analysis of the variation in the motor activity pattern of sleep-wake transition [starting from 120 min before (−120 m) the get-up time (0 m) up to 180 min after (180 m) the get-up time] according to age. The upper panel shows the estimated activity pattern according to age, with different colors depicting the motor activity pattern of sleep-wake transition of participants of different ages. Blue indicates the lowest age, while dark yellow refers to the highest. Activity corresponds to the functional form of raw motor activity data. The lower panel shows the results of the non-parametric permutation F-test. Significant differences can be observed when the solid red line is above the dashed blue line (i.e., the global test of significance, the most conservative).

The results of the FLM analysis on motor SI are reported in Figure 1. The SI appeared to terminate around 70 min after the morning awakening. Considering the most conservative test of significance, higher age was significantly associated with lower motor activity in the first 60 min after the morning awakening.

The potential gender difference in the motor SI has been assessed, but no significant differences have been observed between males’ and females’ motor activity patterns of sleep-wake transition (data not shown).

## 4. Discussion

Regarding the first aim of the present research (i.e., to evaluate the time course of motor SI), we can conclude that motor activity SI in everyday life is dissipated in just over one hour (around 70 min). Overall, this is compatible with what we know about the cortisol awakening response (CAR). However, although deriving from a very high number of observations, this result needs to be replicated before it can be considered conclusive. In particular, this caution is due to the fact that this is the first study to investigate the time course of SI dissipation using motor activity, and therefore no benchmark of any type is currently available.

With reference to the second aim (i.e., to explore the age effects on motor activity SI), the SI phenomenon was much more evident in younger compared to older participants, in line with our expectations. Although younger participants were more active compared to older participants from the get-up time up to 60 min after it, the trajectory of the pattern in the younger participants pointed to a marked decrease in motor activity up to 60 min after the get-up time. In contrast, motor activity levels in the older participants seemed, overall, to be steady over the same time window. We could speculate that this more marked decrease in motor activity in the younger participants within the first 60 min after morning awakening could be interpreted as an epiphenomenon of larger social jetlag [33], commonly associated with negative outcomes as poor sleep quality [34] and daytime sleepiness [35], compared to older participants.

Some limitations of the present research paper must be disclosed. In particular, we considered populations with different social constraints that could reduce their comparability. For example, the main social synchronizer of the school-aged population is the school start time and/or work schedule of their parents, while young adults (mainly university students) and older adults were probably freer to choose when to get up and more generally behave as they wished. However, we think that the naturalistic setting of the present study, as well as the wide age range and the number of nights recorded, could overcome these limitations, providing for the first time the SI dissipation of motor activity.

Finally, we think that our results introduce further interesting questions: can the extent of the SI phenomenon be considered a marker of a person’s well-being? Can we expect a mirrored motor pattern in the transition phase from wakefulness to sleep? We believe that both these questions, alongside the possible role played by chronotype and time-of-day in the modulation of SI, are worthy of further study considering the potential applied implications, especially in the school context and in the promotion of well-being.

## 5. Conclusions

The data presented in this research seem to indicate the usefulness of an evaluation of motor SI, which would seem to have specific dissipation times (around 70 min) that distinguish it from other methods of measurement (e.g., cognitive tasks).

The evaluation of motor SI has several advantages, e.g., it is an easy-to-use, cheap, and ecological assessment.

Further studies will be needed to concretely understand its significance and usefulness (or applicability).

## Data Availability

The data are not publicly available and cannot be shared due to ethical issues.
